# Evaluation of fetal cardiac morphology and function in hypertensive disorders of pregnancy using Fetal HQ technology combined with uterine artery ultrasound parameters

**DOI:** 10.3389/fcvm.2025.1558034

**Published:** 2025-05-02

**Authors:** Lu Wang, Xinghui Fu, Xiaoyu Ren, Yu Guo, Haixia Zhou, Hongli Yue, Chunjing Song, Danhua Zhang, Gang Li, Wenrui Li, Pengjie Zhang

**Affiliations:** ^1^ Department of Ultrasound, The First Affiliated Hospital of Zhengzhou University, Zhengzhou, Henan, China; ^2^ Department of Medical Imaging Technology Teaching and Research Office, Henan Vocational College of Nursing, Anyang, Henan, China; ^3^ Department of Ultrasound, Mengzhou Hospital of Tranditional Chinese Medicine, Jiaozuo, Henan, China; ^4^ Department of Thyroid Surgery, The First Affiliated Hospital of Zhengzhou University, Zhengzhou, China; ^5^ Department of Cardiology, The First Affiliated Hospital of Zhengzhou University, Zhengzhou, China

**Keywords:** Fetal HQ, uterine artery, hypertensive disorders of pregnancy, fetal heart, morphology and function

## Abstract

**Objective:**

Hypertensive disorders of pregnancy (HDP) significantly affect both maternal and fetal health, with uterine artery hemodynamic parameters playing a critical role in assessing fetal well-being, though they do not provide early insights into fetal cardiac function. Fetal Heart Quantification (Fetal HQ) technology offers a non-invasive, highly accurate method for evaluating fetal heart morphology and function, making it a valuable tool for assessing the impact of HDPs on fetal cardiac health.

**Methods:**

This study investigates fetal heart function and morphology in hypertensive disorders of pregnancy (HDPs) using fetal heart quantification (HQ) technology combined with uterine artery ultrasound parameters. A total of 70 normal fetuses and 59 fetuses with HDPs were included, with 30 cases showing normal and 29 cases showing abnormaluterine artery blood flow patterns. Uterine artery hemodynamic parameters (PI, RI, S/D) and fetal echocardiographic parameters (FRAC, FS, GLS, EF, 4CVCirc, GSI) were assessed.

**Results:**

Results showed that in the HDP group with abnormal uterine artery blood flow, PI, RI, and S/D were significantly higher than in both the control and HDP groups with normal blood flow (*P* < 0.05). Right ventricular function, including FRAC, GLS, and FS, was significantly decreased in the HDP group with abnormal blood flow, while 4CVCirc and GSI were significantly different from controls. Left ventricular function showed no significant differences. The area under the ROC curve for predicting fetal heart morphology and function via multiple right ventricular parameters was 0.901, and 0.825 for right heart function.

**Conclusion:**

These findings suggest that the fetal right ventricle is more sensitive to hemodynamic changes in HDP pregnancies, with right heart functional and morphological indicators potentially serving as predictive markers.

## Introduction

Hypertensive disorders of pregnancy (HDP) represent one of the leading causes of morbidity and mortality among both mothers and newborns worldwide. As a common complication during pregnancy, the incidence of HDP is approximately 5%–10% among pregnant women (1). HDP not only endangers maternal health but also poses a range of fetal risks, including intrauterine distress, growth restriction, preterm birth, and, in severe cases, intrauterine fetal death. Research suggests that the core pathophysiological mechanism of HDP involves inadequate placental perfusion and small arterial spasm, which increase the fetal cardiac workload, leading to alterations in fetal hemodynamics and impaired cardiac function (2). Uterine artery hemodynamic parameters are crucial for evaluating the impact of HDPs on fetuses. The pulsatility index (PI), resistance index (RI), and systolic/diastolic velocity ratio (S/D) are key indicators reflecting placental perfusion status. Monitoring uterine artery blood flow enables the assessment of fetal development. Currently, abnormal uterine artery blood flow spectral patterns, such as early diastolic notches, are widely used to predict the adverse effects of HDPs on fetuses (3). The advantages of uterine artery Doppler ultrasound in HDP detection include the following: it is a noninvasive, safe examination method, and analyzing uterine artery blood flow spectral abnormalities can help determine whether the fetus is experiencing intrauterine distress, Uterine artery blood flow primarily reflects the placental-maternal circulation status rather than directly assessing fetal cardiac function. Early compensatory mechanisms of the fetal heart in response to hypoxia (e.g., redistribution of cardiac output, ventricular remodeling) can be detected through parameters obtained from fetal quantitative analysis techniques (such as FRAC and GLS). Fetal Heart Quantification (Fetal HQ) is an innovative technology developed by applying additional techniques for fetal heart assessment based on speckle tracking Imaging, used for non-invasive evaluation of fetal heart morphology and function (4, 5). By analyzing the two-dimensional dynamic four-chamber view (4CV) of the fetal heart, this technique assesses the size, shape, and contractility of the fetal heart. The technology automatically identifies and tracks endocardial motion in real time, dividing the left and right ventricles into 24 equal segments, and uses a simple bidiameter method to measure heart size and shape. It also calculates indicators such as the left ventricular ejection fraction (LVEF), fractional area change (FAC), fractional shortening (FS), and global longitudinal strain (GLS) to evaluate fetal cardiac contractility. A quantitative analysis report is automatically generated, making it feasible to assess overall, longitudinal, and lateral deformation and motion of the ventricles (6–8). This technique is highly sensitive in detecting subclinical cardiac damage, angle independent, easy to perform, and highly accurate and repeatable (9). Fetal HQ provides a new imaging tool with diagnostic value for analyzing the effects of HDP on fetal cardiac morphology and function. Fetal HQ technology demonstrates significant advantages in evaluating cardiac morphology and function in HDP fetuses. Chronic hypoxia caused by placental insufficiency in HDP fetuses often results in increased right ventricular load due to elevated pulmonary circulation resistance. Fetal HQ enables early identification of compensatory right ventricular dilation (enlarged 4CVCirc) and reduced systolic function (decreased GLS), showing higher sensitivity compared to traditional M-mode ultrasound.

This study aimed to assess maternal uterine artery hemodynamic parameters, fetal cardiac function, and morphological indices using fetal heart quantification (Fetal HQ) technology combined with uterine artery blood flow spectral Doppler ultrasound. The goal of this study was to explore the correlation between uterine artery blood flow spectral abnormalities and fetal cardiac function and morphology, as well as to investigate whether these indicators can predict early changes in fetal cardiac function.

## Materials and methods

### Study population

The study included pregnant women who underwent routine fetal ultrasound examinations at the First Affiliated Hospital of Zhengzhou University in China between August 2023 and November 2024. The participants were categorized into two groups on the basis of the diagnostic criteria for hypertensive disorders of pregnancy (HDPs): a control group and a case group (5). The control group consisted of 70 healthy pregnant women, while the case group included 59 women diagnosed with HDP. The case group was further divided into two subgroups on the basis of maternal uterine artery (UA) blood flow spectra: the UA Blood FlowSpectrum Normal Group (30 cases), which showed no significant early diastolic notch in either uterine artery, with a pulsatility index (PI), resistance index (RI), and systolic/diastolic velocity ratio (S/D) all within normal ranges (10), and the UA Blood Flow Spectrum Abnormal Group (29 cases), which exhibited the presence of an early diastolic notch in one or both uterine arteries, along with elevated PI, RI, and S/D values (11). The inclusion criteria included singleton pregnancy, no evident structural heart abnormalities or other congenital malformations in the fetus, gestational age between 24 and 38 weeks, and pregnant women who met the diagnostic criteria for HDP. Informed consent was obtained from all participants and their families, with approval granted by the Ethics Committee (approval number: 2024-KY-1108). The exclusion criteria included pregnant women with conditions that may impact fetal heart morphology and function, such as diabetes or thyroid disorders; fetuses with chromosomal abnormalities or other known genetic disorders (12); pregnant women with systemic inflammatory diseases unrelated to pregnancy-associated infections; and cases where poor image quality during ultrasound imaging prevented the acquisition of accurate data. This study aimed to evaluate the effects of maternal uterine artery blood flow patterns on fetal heart morphology and function via a combination of fetal heart quantification (Fetal HQ) and Doppler ultrasound techniques.

### Ultrasonic examination methods

This study utilized the advanced GE Voluson E10 color Doppler ultrasound system, which is equipped with C1-6/C2-9 probes and specialized Fetal HQ software for fetal heart assessment (GE Healthcare), providing strong technical support for accurate detection. During the uterine artery blood flow evaluation, pregnant women were asked to assume a comfortable supine position. The probe was accurately positioned at the intersection of the bilateral external iliac arteries and the uterine arteries. The probe angle was then meticulously adjusted until the ultrasound beam was as parallel as possible to the direction of blood flow to ensure that the most accurate blood flow signal was obtained (13). Sampling was performed at a point within 1 cm of the uterine artery at the intersection, where no branches were present. After the color Doppler blood flow imaging function was activated, the uterine artery blood flow spectrum was carefully recorded for four consecutive cardiac cycles. During this process, the ultrasound device automatically measures the pulsatility index (PI), resistance index (RI), and systolic/diastolic velocity ratio (S/D) of the uterine artery, with the uterine artery spectrum analyzed according to the 2021 updated ISUOG guidelines (14).

**Figure 1:**
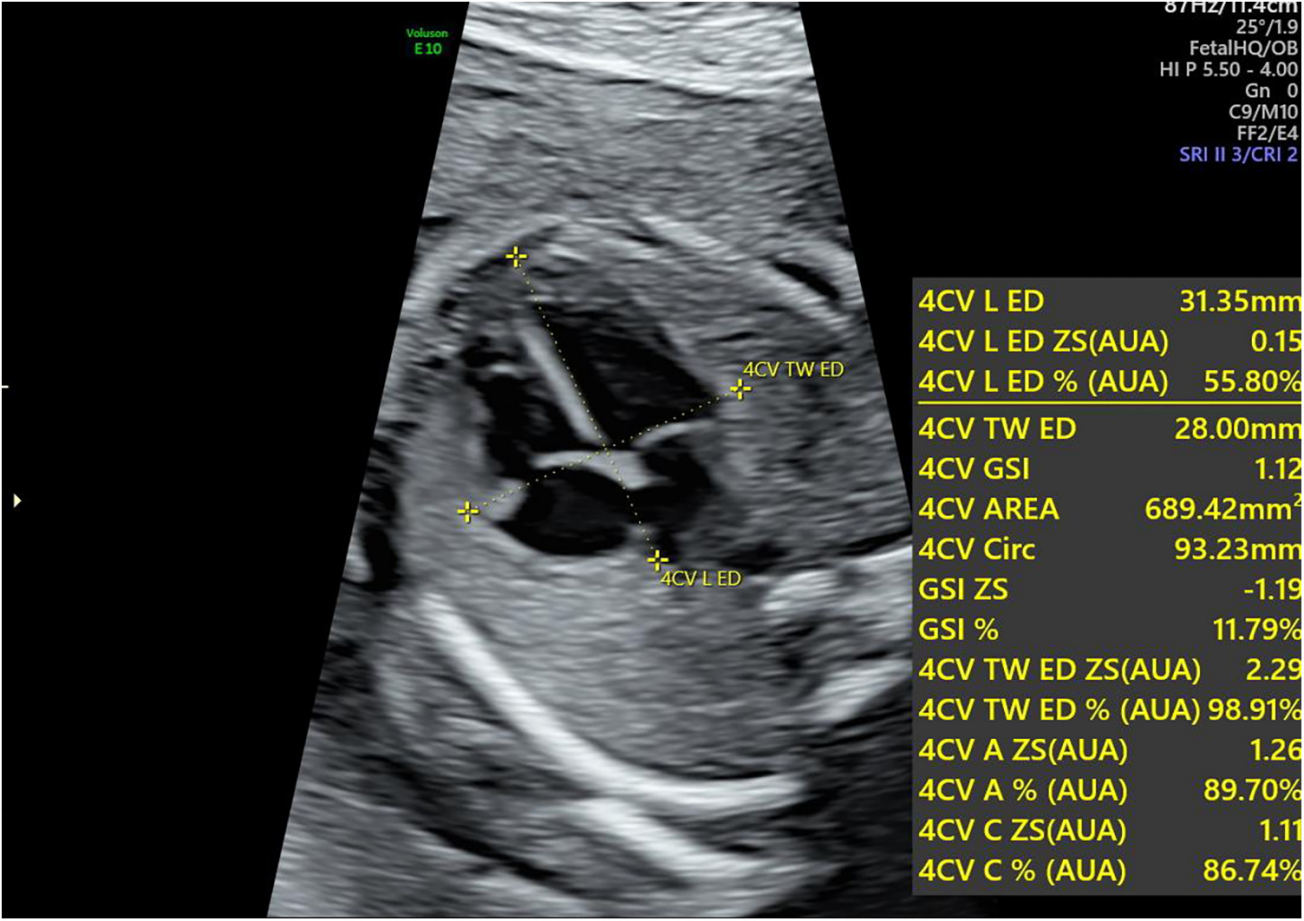
Calculation of the global sphericity index (GSI) in the fetal heart during pregnancy. The longitudinal diameter is measured from the apex to the outer edge of the heart at the end-diastolic phase, whereas the transverse diameter is measured from the left ventricular sidewall to the right ventricular sidewall at the same phase. The GSI is obtained by dividing the longitudinal diameter by the transverse diameter. GSI, global sphericity index.

**Figure 2:**
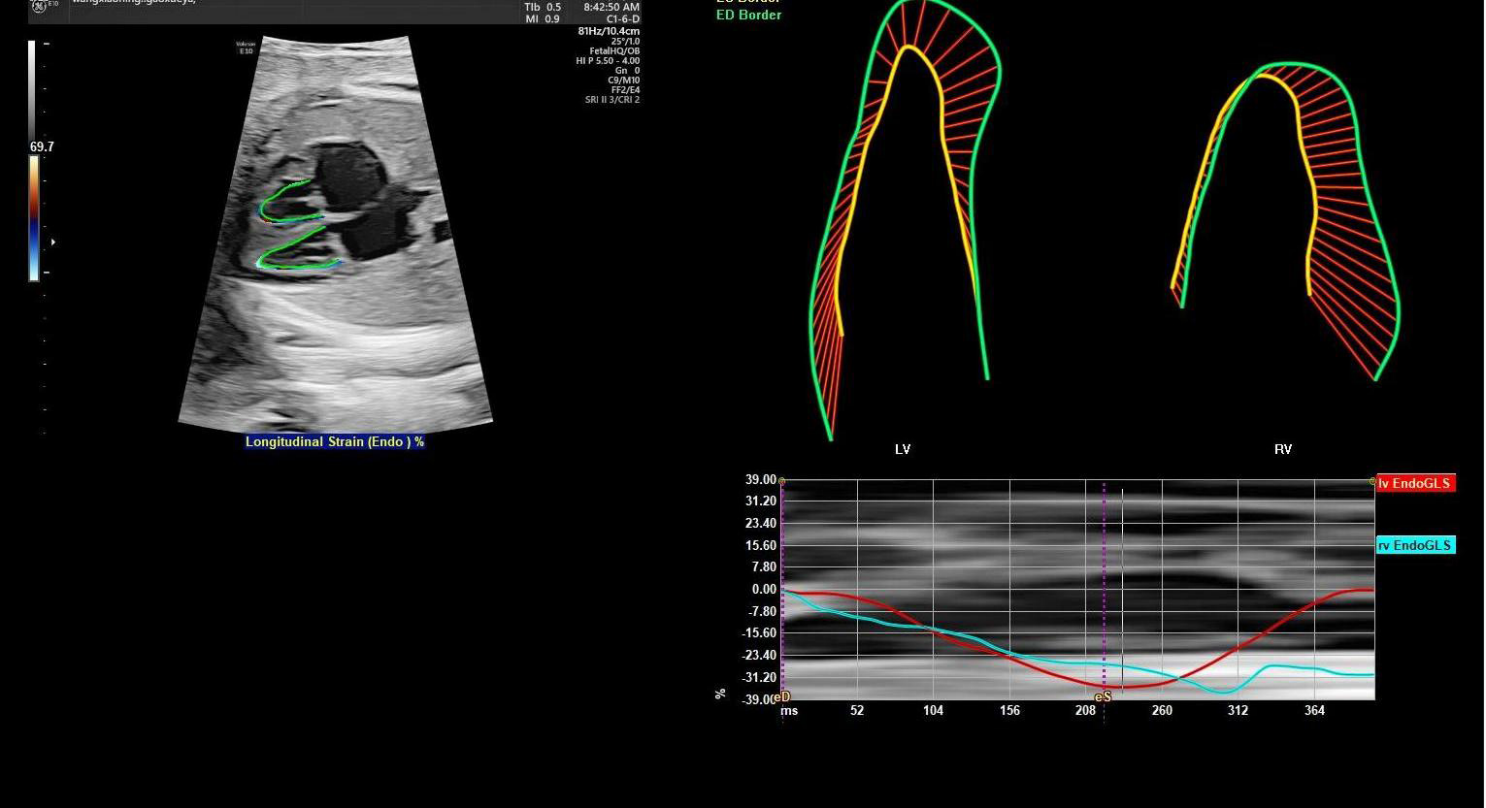
Schematic diagram of two-dimensional speckle tracking of the fetal endocardial motion trajectory. By tracking the motion trajectory of the left and right ventricular endocardium, two-dimensional speckle tracking imaging parameters (GLS, FRAC, EF, and left ventricular 24-segment FS) are obtained. LV, left ventricle; RV, right ventricle; EndoGLS, endocardial global longitudinal strain.

For fetal heart assessment, the standard dynamic four-chamber view (4CV) of he fetal heart was first acquired, ensuring that the image was clear, particularly with the left and right ventricular boundaries and endocardial borders clearly visible, as this forms the basis for subsequent accurate measurement and analysis (15). Fetal HQ software was then used to select a 2–3 s dynamic image, followed by precise marking of the end-diastolic and end-systolic phases to prepare for analysis on the basis of speckle tracking imaging (STI) technology. Using STI technology, semiautomatic tracing of the fetal endocardium was performed to analyze its motion trajectory, allowing the extraction of important parameters reflecting fetal heart function. The specific parameters measured included fractional area change (FRAC), ractional shortening (FS), global longitudinal strain (GLS), left ventricular ejection fraction (EF), and other functional parameters, as well as the global sphericity index (GSI) and ventricular circumference (Circ), for morphological analysis. These parameters provide a comprehensive and detailed understanding of the fetal heart's functional status and morphological structure (as shown in Figures 1, 2).

#### Intra- and inter-observer variability

From the study population, 20 cases were randomly selected. Two ultrasound image reviewers from the same institution performed measurements. After receiving unified training on Fetal HQ ultrasound protocols, one observer repeated measurements within a week, and two different observers conducted separate assessments. This approach likely positively influenced the study results. An ICC value >0.75 indicates good reliability, while >0.8 signifies excellent reliability.

### Statistics

In the data processing and statistical analysis phase, continuous variables are expressed as the means ± standard deviations (means ± SD). For variables that followed a normal distribution with homogeneous variances, independent sample t tests or one-way analysis of variance (ANOVA) were used to compare differences between groups, which effectively assesses the statistical significance of differences across groups. For nonnormally distributed variables, nonparametric tests were applied to ensure the scientific and rational handling of the data. Additionally, receiver operating characteristic (ROC) curves were constructed to evaluate the diagnostic performance of fetal heart function indicators, and the area under the curve (AUC) and its 95% confidence interval (95% CI) were calculated to provide an intuitive understanding of the accuracy and reliability of these indicators in diagnosing fetal heart abnormalities. Furthermore, a multivariate logistic regression model was employed to assess the diagnostic efficacy of the Fetal HQ technique for fetal heart conditions, with a significance level set at *P* < 0.05. This approach aimed to identify which parameters play a key role in the diagnosis of HDP, providing valuable references for clinical diagnosis and treatment.

## Results

### Comparison of uterine artery hemodynamic parameters among the three groups

**Figure 3:**
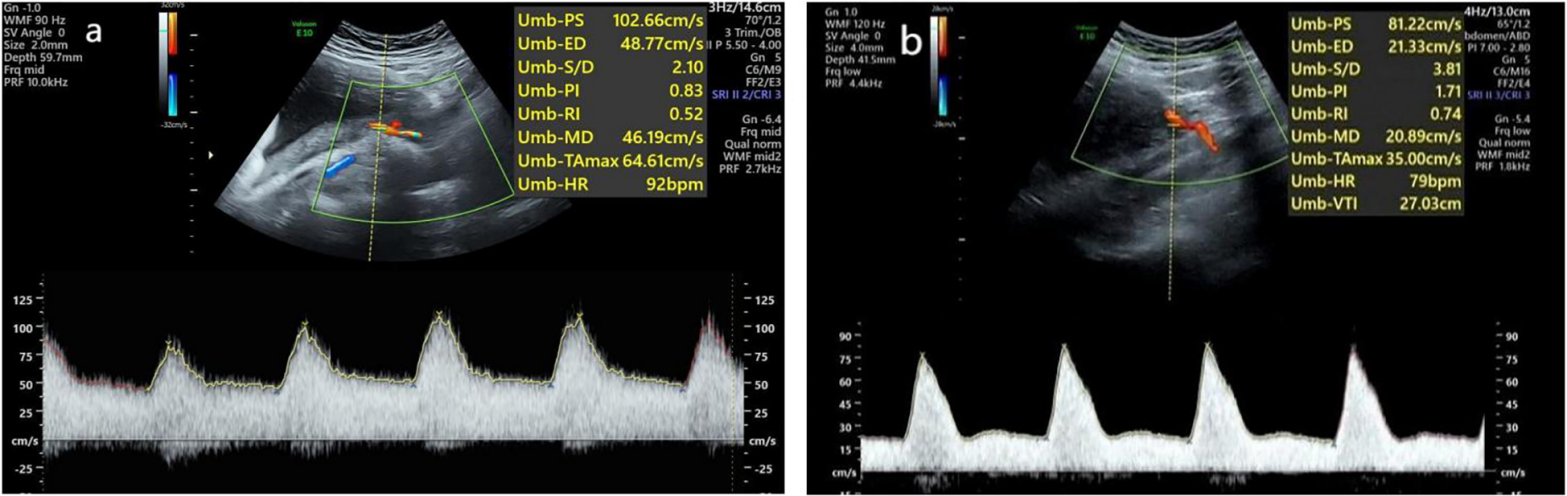
Waveforms from the uterine artery were obtained transabdominally in the second trimester. **(a)** Normal waveform. **(b)** Abnormal waveform.

According to the diagnostic criteria, the HDP patients were divided into a normal uterine artery group (Figure 3a) and an abnormal uterine artery group (Figure 3b). A comparison of the uterine artery hemodynamic parameters (PI, RI, S/D) was conducted to further assess the uterine artery blood flow status. Comparisons among the control group, the uterine artery blood flow spectrum normal HDP group, and the uterine artery abnormal HDP group are presented in Table 1. The results revealed that the pulsatility index (PI), resistance index (RI), and systolic/diastolic velocity ratio (S/D) in the uterine artery blood flow spectrum of the abnormal HDP group were significantly greater than those in the control group and the uterine artery blood flow spectrum of the normal HDP group (*P* < 0.05). In contrast, while the PI, RI, and S/D were slightly greater in the uterine artery blood flow spectrum of the normal HDP group than in that of the control group, the differences were not statistically significant (*P* > 0.05).

**Table 1:** Comparison of uterine artery hemodynamic parameters.

Parameter	Control (Ref.) 95% CI	Negative-HDP 95% CI	Positive-HDP 95% CI	*Sig* ^a^	*Sig* ^b^	*Sig* ^c^
P1	0.62 (0.60, 0.65)	0.64 (0.60, 0.68)	1.43 (1.34, 1.53)	0.404	<0.001	<0.001
R1	0.43 (0.42, 0.45)	0.44 (0.42, 0.46)	0.68 (0.66, 0.70)	0.491	<0.001	<0.001
S/D	1.79 (1.75, 1.83)	1.80 (1.73, 1.86)	3.43 (3.17, 3.69)	0.810	<0.001	<0.001

^a^ Comparison between the control group and the spectrum-negative HDP group.

^b^ Comparison between the control group and the spectrum-positive HDP group.

^c^ Comparison between the spectrum-negative HDP group and the spectrum-positive HDP group.

#### Comparison of fetal heart function and morphological parameters

**Figure 4:**
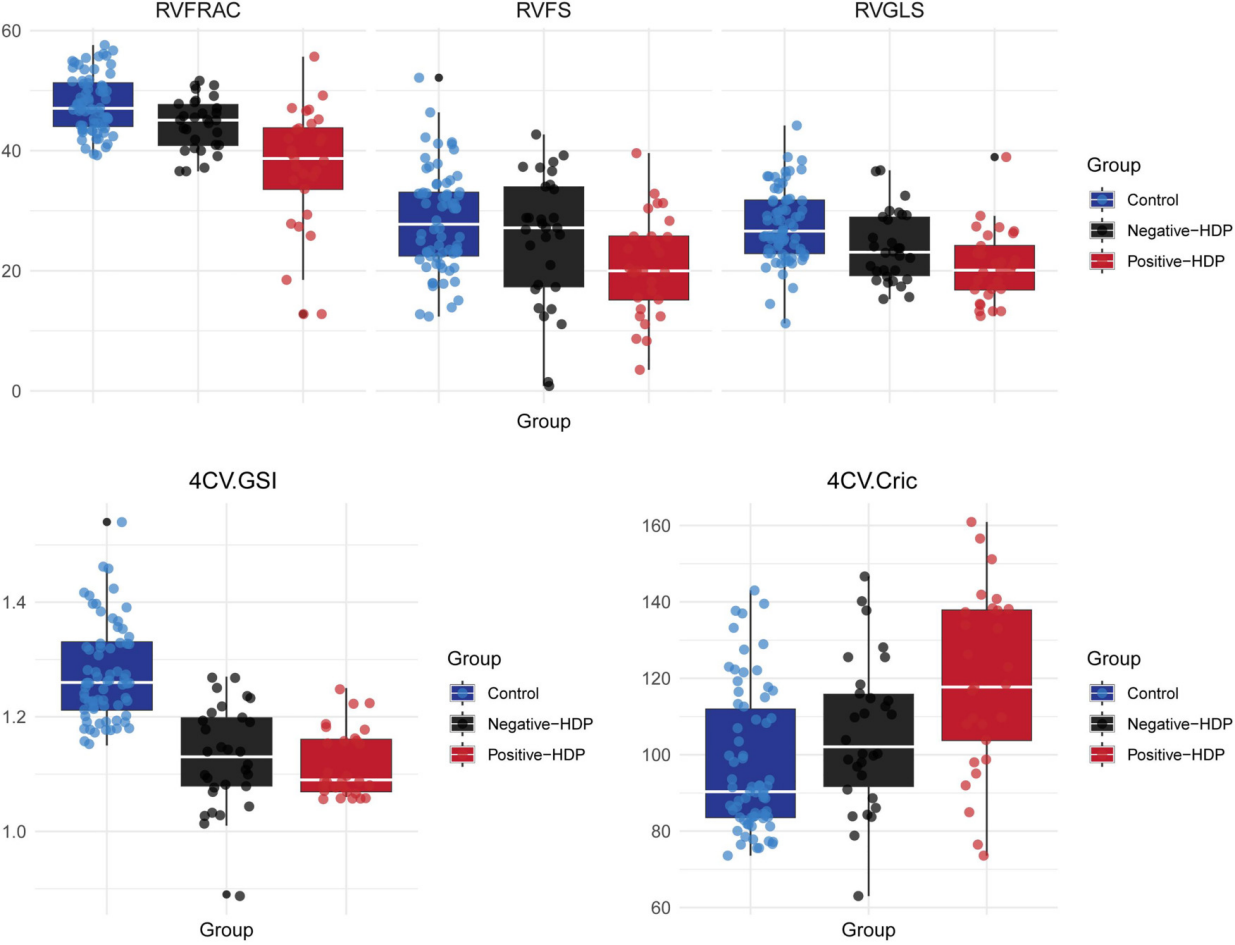
Box plot of right ventricular function and morphological parameters in the HDP group and control group. Control, Control group; Negative-HDP, Uterine artery blood flow spectrum of normal hypertensive disorders in the pregnancy group; Positive-HDP, Uterine artery blood flow spectrum of abnormal hypertensive disorders in the pregnancy group.

We further analyzed the functional and morphological parameters of fetal hearts in pregnant women with hypertensive disorders of pregnancy (HDP) via fetal HQ technology. In the uterine artery blood flow spectrum abnormal HDP group, the righ ventricular fractional area change (FRAC), global longitudinal strain (GLS), and fractional shortening (FS) were significantly lower than those in the control group and the uterine artery blood flow spectrum normal HDP group (*P* < 0.05). The box plots of the right heart function parameters (FRAC, GLS, and FS) further revealed a significant decreasing trend in the abnormal HDP group. In the uterine artery blood flow spectrum of the normal HDP group, FRAC and GLS were lower than those in the control group (*P* < 0.05), but the difference in FS was not statistically significant (*P* > 0.05, Table 2, Figure 4).

**Table 2:** Comparison of fetal heart function parameters.

Indicators	Control (Ref.) 95% CI	Negative-HDP 95% CI	Positive-HDP 95% CI	All samples 95% CI	*Sig* ^a^	*Sig* ^b^	*Sig* ^c^	*Sig* ^d^
LVGLS^e^	28.13 (27.1, 29.16)	28.69 (26.13, 31.25)	27.18 (24.7, 29.66)	28.05 (27.09, 29.01)	0.681	0.476	0.391	0.859
RVGLS^e^	27.54 (26.09, 28.98)	23.87 (21.70, 26.03)	20.74 (18.48, 23.01)	25.16 (24.01, 26.30)	0.006*	<0.001*	0.046*	<0.001*
LVFRAC^e^	49.66 (48.68, 50.64)	48.47 (46.72, 50.23)	47.47 (44.36, 50.58)	48.89 (47.95, 49.84)	0.207	0.178	0.568	0.092
RVFRAC^f^	47.84 (46.71, 48.97)	44.39 (42.78, 46.01)	37.03 (33.1, 40.95)	44.61 (43.28, 45.94)	0.003*	<0.001*	0.001*	<0.001*
LVEF^e^	64.63 (63.8, 65.45)	64.94 (63.27, 66.61)	62.43 (59.52, 65.33)	64.21 (63.34, 65.07)	0.704	0.146	0.131	0.318
LV_FS^f^	30.12 (28.26, 31.98)	29.03 (25.23, 32.84)	25.52 (21.43, 29.6)	28.96 (27.39, 30.54)	0.706	0.067	0.186	0.184
RV_FS1^e^	28.29 (26.31, 30.26)	25.31 (21.28, 29.34)	20.6 (17.43, 23.76)	25.87 (24.23, 27.5)	0.137	<0.001*	0.042	0.001*

Control, control group; Negative-HDP, uterine artery blood flow spectrum of normal hypertensive disorders in the pregnancy group; Positive-HDP, uterine artery blood flow spectrum of abnormal hypertensive disorders in the pregnancy group.

^a^ Comparison of the control group and normal HDP group.

^b^ Comparison of the control group and abnormal HDP group.

^c^ Comparison of the normal HDP group and abnormal HDP group.

^d^ Comparison of the control group and case group.

^e^ Data following a normal distribution; at test was used.

^f^ Data not following a normal distribution; a nonparametric test was used.

*p < 0.05.

For the left heart function parameters (GLS, FRAC, FS, and EF), no significant differences were found among the three groups (*P* > 0.05). However, compared with those in the control group, the mean values of the left heart function parameters were lower in the HDP group. This may be due to the relative advantage of right heart function over left heart function during fetal development, but it also indicates that hypertensive disorders during pregnancy may exert a certain effect on fetal left heart function (Table 2). Owing to the hemodynamic effects of hypertension in pregnant women, increased cardiac load in the fetus not only affects heart function but also causes morphological changes in the heart. The global sphericity index (GSI) and ventricular circumference (4CVCirc) are good indicators of fetal heart morphology. The results revealed that the GSI in both HDP groups was significantly lower than that in the control group (*P* < 0.05), whereas the 4CVCirc in both HDP groups was significantly greater than that in the control group (*P* < 0.05, Table 3). The box plots further illustrated the differences among the three groups (Figure 4). These changes may be due to morphological alterations caused by reduced right heart function in the fetus.

**Table 3:** Comparison of fetal heart morphological parameters.

Indicators	Control (Ref.) 95% CI	Negative-HDP 95% CI	Positive-HDP 95% CI	All samples 95% CI	*Sig* ^a^	*Sig* ^b^	*Sig* ^c^
4CV GSI^d^	1.28 (1.26, 1.30)	1.18 (1.15, 1.21)	1.17 (1.15, 1. 19)	1.23 (1.21, 1.25)	<0.001*	<0.001*	<0.001*
4CV Circ^d^	97.40 (92.95, 1 01.85)	105.42 (98.16, 112.68)	118.61 (109.79, 127.42)	104.03 (100.28, 107.78)	0.024*	<0.001*	<0.001*

Control, control group; Negative-HDP, uterine artery blood flow spectrum of normal hypertensive disorders in the pregnancy group; Positive-HDP, uterine artery blood flow spectrum of abnormal hypertensive disorders in the pregnancy group.

^a^ Comparison of the control group and normal HDP group.

^b^ Comparison of the control group and abnormal HDP group.

^c^ Comparison of the control group and case group.

^d^ Data not following anormal distribution; a nonparametric test was used.

*p < 0.05.

### Diagnostic efficacy analysis

**Figure 5:**
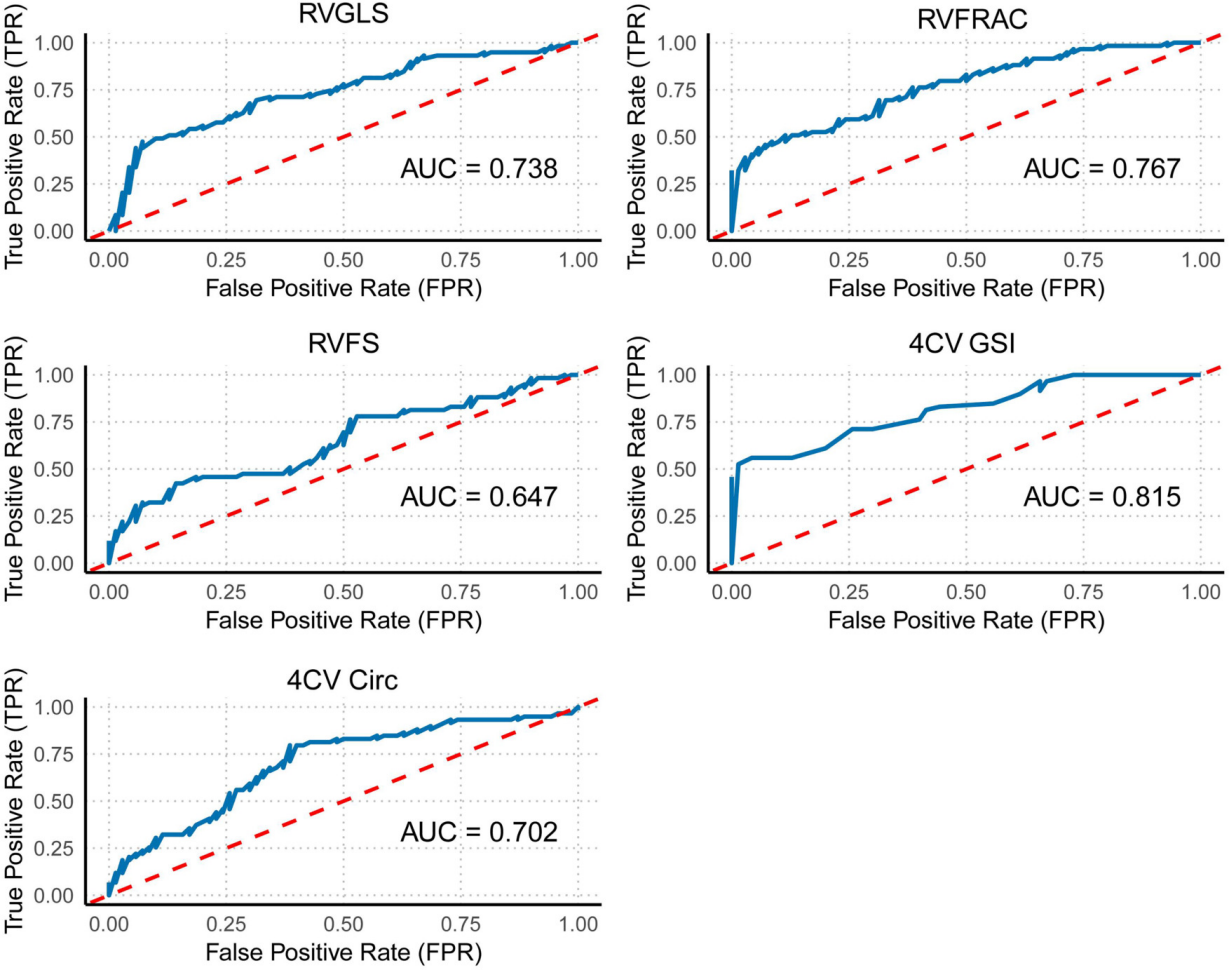
ROC curve of individual right ventricular indicators. This figure shows the ROC curves for individual right ventricular function indicators (GLS, FRAC, and FS) and overall cardiac morphological indicators (GSI and Circ).

The advantage of fetal HQ technology lies in its ability to obtain multiple fetal cardiac functions and morphological parameters in a single scan. While individual parameters can reflect cardiac changes to a certain extent, the integration of multiple parameters offers a more comprehensive and accurate assessment of fetal cardiac function and morphology, especially in the early stages of disease. The diagnostic efficacy of individual indicators is shown in Figure 5.

Receiver operating characteristic (ROC) curve analysis of fetal cardiac function and morphological indicators (FRAC, GLS, FS, GSI, and 4CVCirc) revealed the following results: among the right ventricular function and morphological indicators, the area under the curve (AUC) for distinguishing between the control and case groups ranged from 0.647–0.815. Specifically, the 4CVGSI had the highest AUC at 0.815. Except for RVFS, which had a relatively low AUC value (AUC = 0.647), the other indicators showed good discriminatory ability when detecting right ventricular dysfunction, with RVGLS (AUC = 0.738), RVFRAC (AUC = 0.767), and 4CVCirc (AUC = 0.702) demonstrating strong diagnostic value (Table 4, Figure 5).

**Table 4:** AUC and 95% CI for each indicator.

Variables	AUC 95% CI	*P*
RVGLS	0.738 (0.651, 0.826)	<0.001
RVFRAC	0.767 (0.686, 0.848)	<0.001
RVFS	0.647 (0.550, 0.743)	0.004
4CV GSI	0.815 (0.742, 0.888)	<0.001
4CV Circ	0.702 (0.611, 0.793)	<0.001
Logistic-LR	0.901 (0.848, 0.954)	<0.001
Logistic-Enter	0.825 (0.753, 0.897)	<0.001

**Figure 6:**
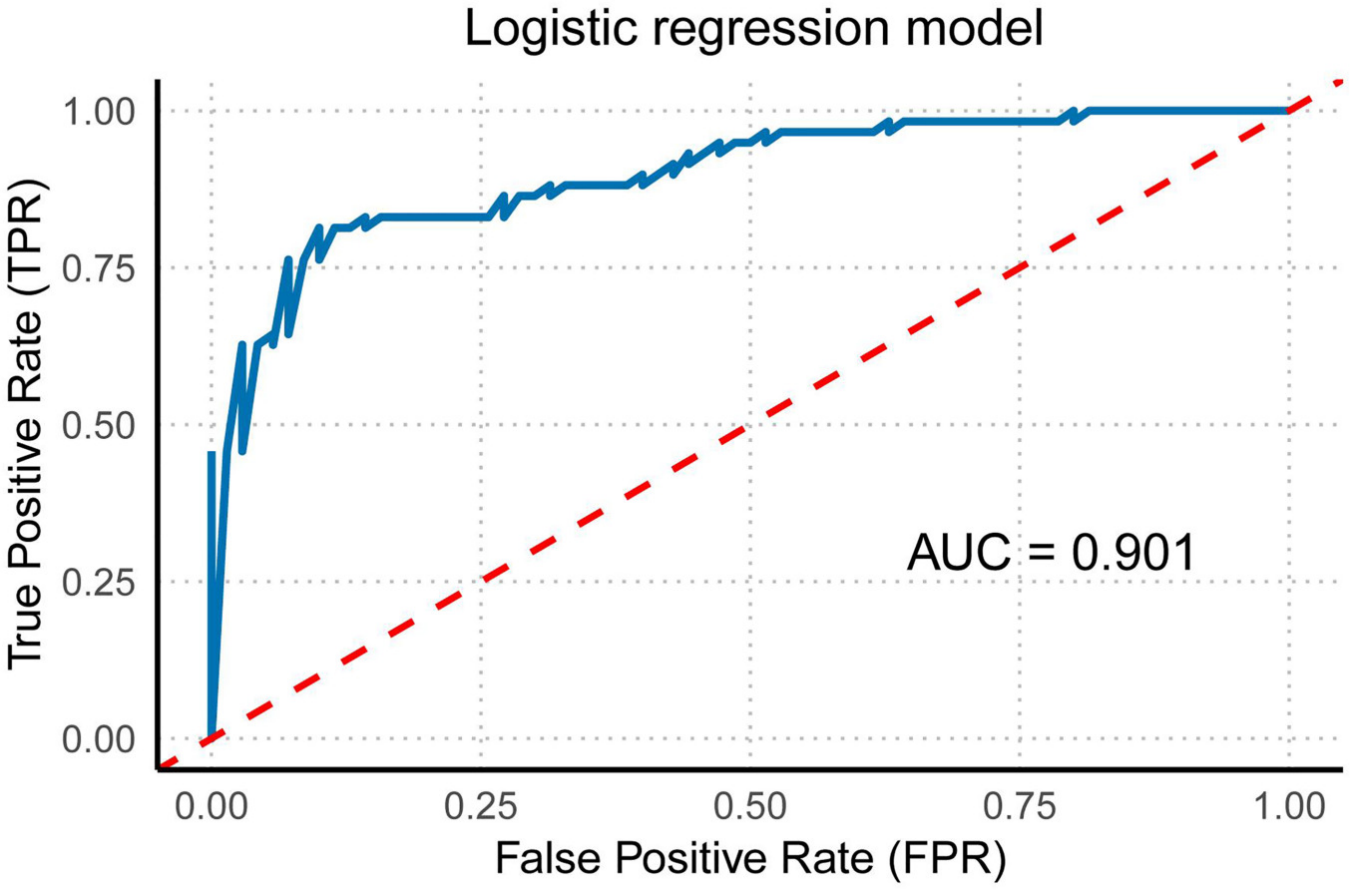
Multivariate regression model of right ventricular function and morphology. This figure illustrates the multivariate regression model of right ventricular function and morphology (including RVGLS, RVFRAC, RVFS, 4CVGSI, and 4CVCirc) and its corresponding ROC curve with the area under the curve (AUC).

Using the Forward method for multivariate regression analysis, multiple fetal cardiac quantitative indicators (GLS, FRAC, RVFS, GSI, 4CVCirc) were analyzed. The results showed that the model incorporating RVGLS, RVFRAC, RVFS, 4CV GSI, and 4CV Circ achieved an ROC curve AUC of 0.901 (95% CI: 0.848–0.954). This confirms the diagnostic efficacy of these indicators for fetal cardiac impairment in HDP. A combined multi-indicator ROC analysis revealed that the integrated model exhibited higher sensitivity and specificity in distinguishing HDP fetuses with cardiac dysfunction from controls, with significantly superior AUC compared to individual indicators (Figure 6).

**Figure 7:**
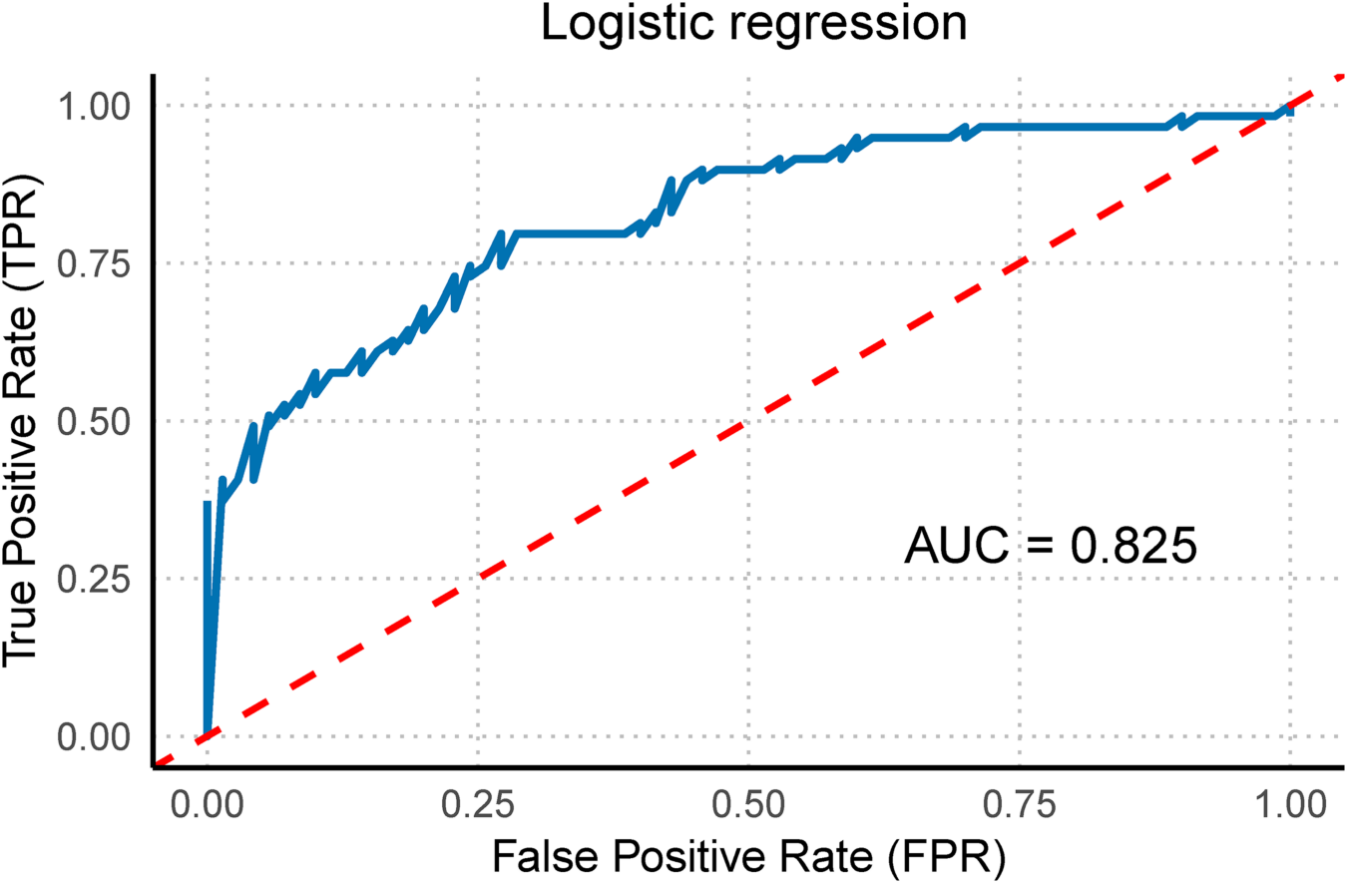
Right ventricular function multivariate regression model. This figure illustrates the multivariate regression model of right ventricular function (including RVGLS, RVFRAC, and RVFS) and its corresponding ROC curve with the area under the curve (AUC).

Owing to the unique hemodynamic changes that occur during fetal development, the right heart plays a dominant role in fetal circulation. Therefore, evaluating right ventricular function is particularly important. When right ventricular function indicators (RVGLS, RVFRAC, and RVFS) were included in the model and stepwise regression analysis was conducted, the results revealed that this model had a strong ability to distinguish between the case and control groups. The AUC of the ROC curve was 0.825 (95% CI: 0.753–0.897), demonstrating excellent diagnostic value (Figure 7). Thus, this technology not only allows for a comprehensive evaluation of overall fetal cardiac changes but also enables the assessment of subtle changes in specific heart chambers. This information is crucial for identifying the potential risks posed by hypertensive disorders of pregnancy to the fetal heart, providing valuable diagnostic information for early clinical intervention.

### Reproducibility

All parameters demonstrated excellent intra- and inter-observer reliability (ICC >0.8). These results indicate that Fetal HQ technology offers high repeatability and ease of use. Skilled sonographers can typically complete fetal cardiac analysis within 5 min. The semi-automatic endocardial analysis software further ensures strong reproducibility. In summary, Fetal HQ provides an accurate and effective method for assessing fetal cardiac function independent of apical four-chamber view orientation, serving as a valuable new diagnostic tool (Table 5).

**Table 5:** Intra-observer and inter-observer correlation.

Intra-observer Inter-observer				
Measurements	ICC 95% confidence interval		ICC 95% confidence interval	
LV-GLS	0.970	0.910–0.990	0.958	0.880–0.986
RV-GLS	0.916	0.800–0.966	0.926	0.823–0.970
LV-FAC	0.944	0.865–0.978	0.963	0.908–0.985
RV-FAC	0.965	0.913–0.986	0.973	0.934–0.989
4CV-GSI	0.984	0.961–0.994	0.989	0.972–0.996

ICC, intra-class correlation coefficients; GSI, global sphericity index of LV left ventricle and RV right ventricle; FAC, fractional area change of LV left ventricle and RV right ventricle; 4CV, four-chamber view.

## Discussion

Under physiological conditions, the subendocardial myocardium receives a rich blood supply, and when ischemia or hypoxia occurs, the endocardium is the first to be damaged. Therefore, detecting endocardial motion can reflect changes in fetal heart function early in life (16–18). This study employed fetal cardiac quantification technology (Fetal HQ) in combination with the maternal uterine artery blood flow spectrum to explore the impact of hypertensive disorders of pregnancy (HDP) on fetal heart function (19). The study revealed that right heart function indicators, such as global longitudinal strain(GLS), fractional area change (FRAC), and fractional shortening (FS), were reduced in fetuses with HDP. Moreover, these right heart function indicators decreased more significantly as uterine artery abnormalities (such as early diastolic notching, increased PI, and RI) appeared (*P* < 0.05). In comparison, although left heart function indicators also showed some decline, no statistically significant differences were observed (*P* > 0.05), indicating that the right ventricle is more sensitive to hemodynamic changes (20, 21).

This study suggests that abnormal uterine artery blood flow spectra are closely linked to impaired fetal cardiac function. Mechanistically, abnormal uterine artery blood flow educes placental perfusion, leading to chronic fetal hypoxia (22). Abnormal ductus venosus hemodynamics increases right ventricular preload, causing right ventricular dilation and myocardial remodeling (23). Reduced FRAC reflects insufficient right ventricular filling due to decreased venous return; decreased GLS indicates hypoxia-induced cardiomyocyte apoptosis, fibrosis, and diminished longitudinal contractility; and reduced FS signifies excessive right ventricular afterload beyond compensatory capacity (24). Declines in these parameters predict reduced fetal cardiac output, potentially progressing to heart failure, IUGR, or intrauterine demise. Early detection of right ventricular dysfunction can thus guide clinical interventions. These findings are consistent with those of Day et al., who reported that fetal right ventricular systolic function is subclinically impaired (25), and the feasibility of using GLS and FS as indicators during the fetal period has been confirmed (26, 27).

From a mechanistic perspective, HDP is characterized by small artery spasm, increased vascular resistance, and insufficient placental perfusion, which leads to fetal oxygen deficiency. This, in turn, triggers the “brain-protective effect” to prioritize blood supply to the brain and heart, but the increased cardiac load directly interferes with myocardial function (28). Studies also suggest that the compensatory load on the fetal right ventricle is significantly greater than that on the left ventricle and that early changes in right heart function may precede abnormalities in overall cardiac morphology, highlighting the heightened sensitivity of the right ventricle to abnormal blood flow perfusion (29).

This study revealed that the pulsatility index (PI), resistance index (RI), and systolic-to-diastolic velocity ratio (S/D) in the abnormal uterine artery flow spectrum HDP group were significantly greater than those in the control group and the normal flow spectrum HDP group (*P* < 0.05). In the uterine artery flow spectrum abnormal HDP group, indicators of right heart function, including FRAC, GLS, FS, 4CVGSI, and 4CVCirc, were significantly different from those in the control group and normal flow spectrum HDP group (*P* < 0.05). In contrast, in the normal flow spectrum HDP group, only FRAC and GLS were significantly lower than those in the control group (*P* < 0.05), whereas FS was not significantly different (*P* > 0.05). These findings further indicate that uterine artery blood flow abnormalities in HDPs are highly correlated with fetal heart function impairment and that early diastolic notching, as an independent predictor of preeclampsia and poor fetal prognosis, is widely used in clinical practice. Combined with uterine artery blood flow changes, it helps monitor the progression of HDP and provides key references for early intervention in fetal heart function abnormalities (30).

Compared with previous studies, while the left heart function indicators in this study showed a downward trend, they did not reach statistical significance (*P* > 0.05). This may be due to the small sample size, differences in the inclusion criteria or grouping methods. For example, some previous studies included pregnant women with additional complications, while this study strictly excluded other interfering factors. Additionally, changes in left heart function may only become apparent in later stages of the disease, whereas this study focused on early changes in fetal heart function. Furthermore, this study explores the mechanisms of fetal heart damage by combining maternal uterine artery blood flow parameters, highlighting the importance of UA blood flow abnormalities and fetal HQ technology in the assessment and prediction of HDP, which aligns with earlier research findings. This combined analysis provides a new perspective for comprehensively understanding the impact of HDPs on fetal heart function (31–33).

Fetal HQ technology, with its speckle tracking imaging technique, precisely quantifies fetal endocardial motion. Its 24-segment ventricular partition design allows for detailed analysis of cardiac morphology and functional parameters, overcoming the limitations of traditional ultrasound and thereby improving assessment sensitivity and accuracy. This provides a new means for the early detection of subclinical fetal heart damage (34). Unlike conventional 2D ultrasound and color Doppler flow technology, fetal HQ technology is not affected by the image angle and integrate multiple parameters to comprehensively evaluate cardiac morphological and functional changes. As a result, it can detect fetal heart function abnormalities in a timely manner, which is crucial in the study of gestational hypertensive diseases and provides valuable information for clinical intervention strategies. To validate the diagnostic efficacy of cardiac systolic function and morphological indicators for HDP-related fetal cardiac impairment, ROC curves were plotted for individual parameters. All indicators showed discriminative power for HDP. The multi-indicator model demonstrated higher sensitivity, specificity, and AUC compared to single parameters. However, the relatively small sample size limits this study to preliminary validation of feasibility. Future large-scale, multicenter prospective studies could standardize application criteria and expand its use to fetal cardiac assessment in other pregnancy complications, advancing obstetric imaging diagnostics.

From a clinical perspective, this study confirmed the value of fetal HQ technology in assessing fetal heart dysfunction in HDP patients. By utilizing simple four-chamber view imaging, it can be used to analyze fetal heart morphology and function quantitatively, providing a reliable tool for the early detection of right heart dysfunction in fetuses. These findings have significant implications for the early intervention and management of high-risk pregnancies in HDP patients (35). Since HDP is prone to cause adverse pregnancy outcomes such as fetal growth restriction and intrauterine distress, early assessment of fetal heart function can help predict fetal prognosis. By jointly evaluating maternal uterine artery blood flow parameters and fetal heart function indicators, this study provides scientific evidence for the development of personalized intervention strategies, thereby reducing the risk of HDP-related maternal and fetal complications.

However, there are several limitations in this study. During image acquisition, fetal position and fetal movements may lead to echo loss in the apex region, especially when the endocardial motion in the apical segment is captured. This can result in measurement deviations, affecting the accuracy of certain parameters, such as the GSI (36). Furthermore, as this is a single-center retrospective study with a relatively small sample size, fully encompassing the various pathological manifestations of HDP is difficult. Future research should aim to expand the sample size and conduct multicenter prospective studies to increase the generalizability and applicability of the findings. Despite the significant value of Fetal HQ technology in assessing fetal heart function and morphology, research on the application of speckle tracking technology in pregnancy complications (such as gestational diabetes, fetal growth restriction, and preeclampsia) remains limited. More clinical studies are needed to explore its potential applications across different gestational periods and disease states (37, 38).

It has been suggested that umbilical blood flow abnormalities are a key intermediate link in fetal cardiac insufficiency caused by HDP, which mediates myocardial remodeling and dysfunction by increasing right heart load and triggering hypoxic-oxidative stress. The significant decrease in right heart GLS and FRAC in the HDP uterine artery anomaly group in the present study may be consistent with the increase in right heart load due to umbilical blood flow anomalies, and future integration of multidimensional hemodynamic parameters is needed to comprehensively elucidate the potential mechanisms of fetal cardiac injury in HDP. Abnormal uterine arterial blood flow has been found to be associated with low Apgar score and risk of neonatal ICU admission (39), Data from the postpartum and neonatal stages have not been followed up in the present study. Although fetal HQ techniques showed that right heart function parameters (e.g., GLS, FRAC) were significantly associated with HDP, there is a lack of data on the cardiac status or clinical outcomes of the neonates. In the future, we will combine the postpartum data to more fully assess. The impact of abnormal fetal cardiac function on neonatal health will be more comprehensively assessed in conjunction with postnatal data in the future to provide a scientific basis for the development of individualized intervention strategies.

## Conclusions

Fetal cardiac quantification technology (Fetal HQ) combined with uterine artery blood flow assessment can sensitively and accurately reflect the impact of hypertensive disorders of pregnancy (HDP) on fetal heart function and morphology. Right heart function parameters, such as global longitudinal strain (GLS), fractional area change (FRAC), and fractional shortening(FS), as well as morphological indicators such as the global sphericity index (GSI) and four-chamber circumference(4CVCirc), are of significant diagnostic value in assessing early fetal heart abnormalities in HDP patients and can be used as key indicators in clinical evaluations. This study, by jointly assessing fetal heart function and maternal uterine artery blood flow parameters, provides not only a reliable imaging basis for the early detection of fetal heart dysfunction in HDP patients but also scientific support for prenatal management and intervention strategies for HDP patients.

## Data Availability

The original contributions presented in the study are included in the article/Supplementary Material, further inquiries can be directed to the corresponding author.
